# Utility of Mass SARS-CoV-2 Testing of Asymptomatic Patients Before Ambulatory and Inpatient Preplanned Procedures Requiring Moderate Sedation or General Anesthesia

**DOI:** 10.1001/jamanetworkopen.2021.14526

**Published:** 2021-06-25

**Authors:** Scott C. Roberts, David R. Peaper, L. Scott Sussman, Richard A. Martinello, Christian M. Pettker

**Affiliations:** 1 Department of Internal Medicine, Yale School of Medicine, New Haven, Connecticut; 2 Department of Laboratory Medicine, Yale School of Medicine, New Haven, Connecticut; 3 Clinical Redesign, Yale New Haven Health, New Haven, Connecticut; 4 Department of Obstetrics, Gynecology, and Reproductive Sciences, Yale School of Medicine, New Haven, Connecticut

## Abstract

This quality improvement study describes the results of a mass preprocedure SARS-CoV-2 testing strategy during a period of high community transmission in Connecticut.

## Introduction

Systematic management of procedures during the COVID-19 crisis is a priority to (1) detect and care appropriately for patients with COVID-19, (2) prevent outbreaks, and (3) safely maintain routine health care activities. SARS-CoV-2 presents unique challenges, as an estimated 50% of infections occur through asymptomatic transmission, and clinical screening may miss contagious patients.^1,2^ These factors complicate settings where aerosol-generating procedures (AGPs) are performed, potentially exposing health care personnel (HCP) to SARS-CoV-2 transmission. Furthermore, patients with COVID-19 have increased risks of postprocedural complications, and current guidelines suggest that testing asymptomatic patients prior to preplanned procedures may help to mitigate these risks.^3^ We describe our mass preprocedure SARS-CoV-2 nucleic acid amplification testing (NAAT) during a period of high community transmission.^4^

## Methods

This quality improvement project was exempt from institutional review board review and informed consent, per Yale University institutional review board policy. We followed the Standards for Quality Improvement Reporting Excellence (SQUIRE) reporting guideline.^5^

Yale New Haven Health System tested asymptomatic patients within 3 days of select ambulatory and inpatient procedures (AGPs and procedures requiring moderate sedation or general anesthesia) from August 1 to November 30, 2020. The primary outcome was a positive preprocedure SARS-CoV-2 test. Patients received NAAT on nasopharyngeal or deep midturbinate nasal swabs on a US Food and Drug Administration–authorized platform. Patients testing positive in the prior 90 days were not required to undergo repeated testing but could be tested at clinician’s discretion. To evaluate for missed infections, we reviewed results up to 7 days after the preprocedure test when available. HCP wore respirators (or equivalent), eye protection, gowns, and gloves for all AGPs regardless of patient COVID-19 status. Data were collected and analyzed in Excel version 16.49 (Microsoft Corp).

## Results

**Figure 1.**
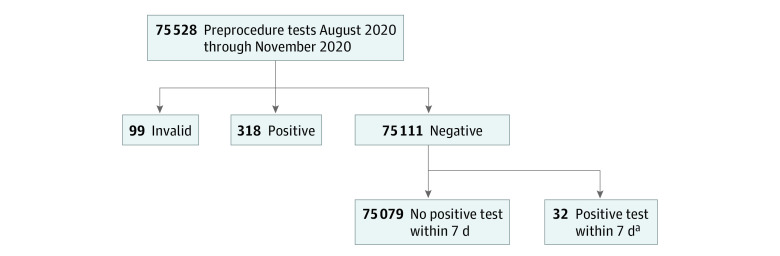
Flow Diagram of Preprocedure Tests Performed ^a^A total of 32 positive specimens from 31 patients were positive within 7 days of an initial negative preprocedure test result. Three patients were positive more than 100 days and 1 patient 63 days before the positive preprocedure test, suggesting low-level persistent RNA positivity.

**Figure 2.**
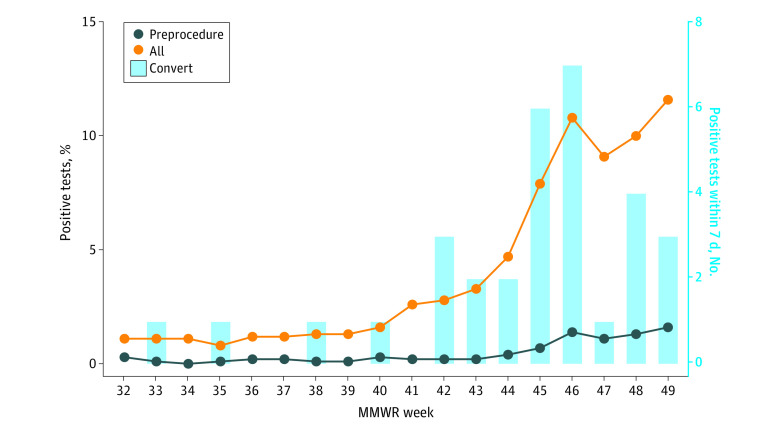
Positive Preprocedural vs Overall Tests Overall systemwide positive tests compared with preprocedure positive tests, with initial negative followed by a positive conversion within 7 days noted. Week 47 reflects the Thanksgiving holiday, when less testing occurred. MMWR indicates *Morbidity and Mortality Weekly Report*.

A total of 75 528 preprocedure tests were performed. The median (interquartile range [IQR]) age of participants was 59 (44-69) years and 33 746 (44.7%) were male patients. A total of 318 (0.4%) tested positive (Figure 1). The median (IQR) test turnaround time was 7.8 (6.5-9.4) hours. In 32 cases with 31 patients, preprocedure testing was negative, but a positive result occurred within 7 days (median [IQR], 4.0 [3.0-5.3] days) (Figure 2). Fourteen of these patients (45.2%) developed symptoms confirming COVID-19, while 17 (54.8%) did not. Seven patients (22.6%) had prior positive testing, a median (IQR) of 134.0 (123.8-152.5) days prior to the positive result. We obtained cycle threshold values for 2 patients (6.5%), both of which were consistent with low-level RNA presence.^6^ Six patients (19.4%) confirmed positive household contacts after being informed of their positive test. During the evaluation, community SARS-CoV-2 rates ranged between 5 and 179 daily positive tests per 100 000 individuals.^4^

## Discussion

With preprocedure testing, we detected asymptomatic patients with SARS-CoV-2 who were missed by clinical screening alone. Testing programs like this one can reduce potential transmission events while providing an additional layer of HCP safety. Postprocedural infectious complications of COVID-19 in this higher-risk population are also mitigated when COVID-19 status is known and procedural delay is possible. Additionally, the resource burden of performing procedures using COVID-19 precautions is reduced.

The optimal timing for preprocedural testing is unknown. In rare circumstances, patients who initially tested negative later tested positive, reflecting either low viral burden early in the disease course, false-negative laboratory errors, suboptimal specimen collection, nosocomial transmission, or variable viral particle shedding in an individual long recovered from COVID-19.^6^ While the incidence of such conversions was low, occurrences were more common during higher community prevalence. Some patients had known, although previously undisclosed, COVID-19 household contacts. Obtaining preprocedure SARS-CoV-2 rapid antigen tests may aid in detection while obviating the logistic challenges of NAAT.

Limitations of this study include underreporting bias; few patients had indications for postprocedure testing, and we may not have identified all patients testing positive after the initial preprocedure negative test result. While institutions should weigh the burden of preprocedure testing against managing patients with occult infections, universal preprocedure SARS-CoV-2 testing should be considered.
